# Supplementary figures and images for: Antimicrobial Resistance and Comparative Genomic Analysis of Elizabethkingia anophelis subsp. endophytica Isolated from Raw Milk
Source: Antibiotics (Basel). 2022 May 12;11(5):648. doi: 10.3390/antibiotics11050648 (PMC9137776; doi:10.3390/antibiotics11050648)

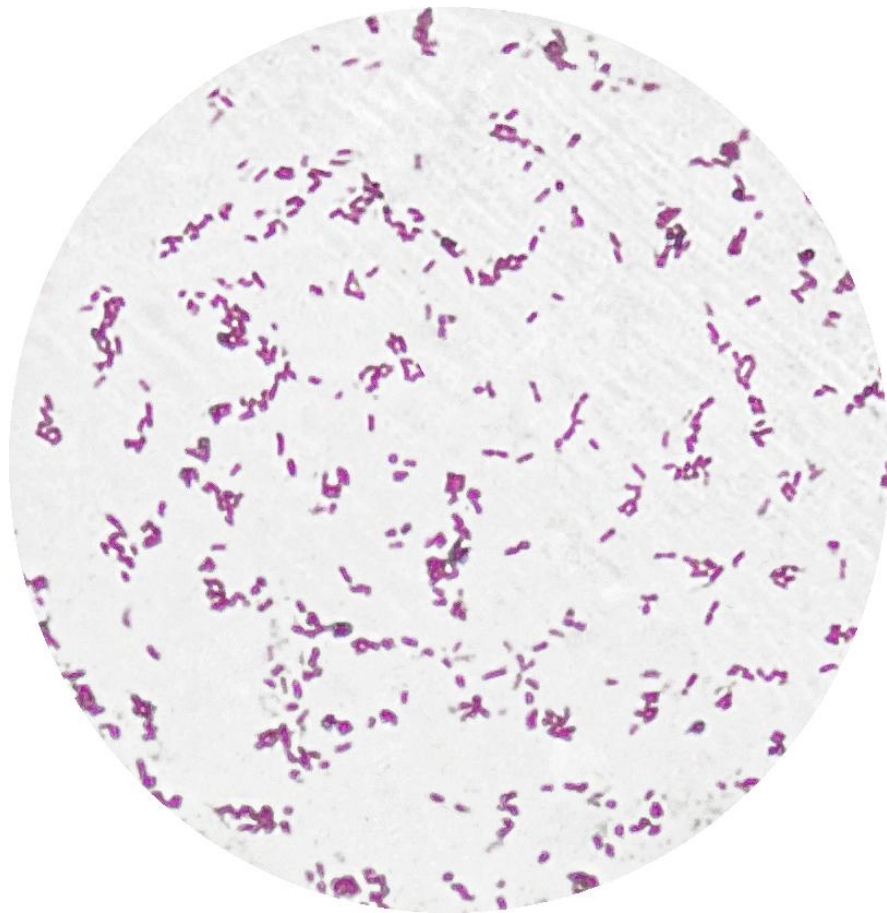

**Figure S1.** Gram-stained smear of pure *Elizabethkingia anophelis* ML-44 culture.

Supplement: Supplementary file 1 [file antibiotics-11-00648-s001.zip › Figure S1.pdf]
